# Early Environment and Neurobehavioral Development Predict Adult Temperament Clusters

**DOI:** 10.1371/journal.pone.0038065

**Published:** 2012-07-18

**Authors:** Eliza Congdon, Susan Service, Jaana Wessman, Jouni K. Seppänen, Stefan Schönauer, Jouko Miettunen, Hannu Turunen, Markku Koiranen, Matti Joukamaa, Marjo-Riitta Järvelin, Leena Peltonen, Juha Veijola, Heikki Mannila, Tiina Paunio, Nelson B. Freimer

**Affiliations:** 1 University of California Los Angeles Center for Neurobehavioral Genetics, Semel Institute for Neuroscience and Human Behavior, University of California Los Angeles, Los Angeles, California, United States of America; 2 Helsinki Institute for Information Technology and Department of Computer Science, University of Helsinki, Helsinki, Finland; 3 Institute for Molecular Medicine Finland and National Institute for Health and Welfare, Helsinki, Finland; 4 Helsinki Institute for Information Technology and Aalto University, Espoo, Finland; 5 Department of Psychiatry, University of Oulu and Oulu University Hospital, Oulu, Finland; 6 Institute of Health Sciences, University of Oulu, Oulu, Finland; 7 Tampere School of Public Health, University of Tampere, and Psychiatric Department, Tampere University Hospital, Tampere, Finland; 8 Department of Epidemiology and Biostatistics, School of Public Health, Imperial College, Faculty of Medicine, London, United Kingdom; 9 Biocenter Oulu, University of Oulu, Oulu, Finland; 10 National Institute of Health and Welfare, Oulu, Finland; 11 Wellcome Trust Sanger Institute, Hinxton, Cambridge, United Kingdom; 12 Department of Medical Genetics, University of Helsinki, Helsinki, Finland; Catholic University of Sacred Heart of Rome, Italy

## Abstract

**Background:**

Investigation of the environmental influences on human behavioral phenotypes is important for our understanding of the causation of psychiatric disorders. However, there are complexities associated with the assessment of environmental influences on behavior.

**Methods/Principal Findings:**

We conducted a series of analyses using a prospective, longitudinal study of a nationally representative birth cohort from Finland (the Northern Finland 1966 Birth Cohort). Participants included a total of 3,761 male and female cohort members who were living in Finland at the age of 16 years and who had complete temperament scores. Our initial analyses (Wessman et al., *in press*) provide evidence in support of four stable and robust temperament clusters. Using these temperament clusters, as well as independent temperament dimensions for comparison, we conducted a data-driven analysis to assess the influence of a broad set of life course measures, assessed pre-natally, in infancy, and during adolescence, on adult temperament.

**Results:**

Measures of early environment, neurobehavioral development, and adolescent behavior significantly predict adult temperament, classified by both cluster membership and temperament dimensions. Specifically, our results suggest that a relatively consistent set of life course measures are associated with adult temperament profiles, including maternal education, characteristics of the family’s location and residence, adolescent academic performance, and adolescent smoking.

**Conclusions:**

Our finding that a consistent set of life course measures predict temperament clusters indicate that these clusters represent distinct developmental temperament trajectories and that information about a subset of life course measures has implications for adult health outcomes.

## Introduction

Understanding the causation of psychiatric disorders will require dissection of the specific genetic and environmental determinants of disease susceptibility. Yet the two components of this task differ enormously in their feasibility. The genetic variations contributing to such susceptibility, although mostly still unknown, are knowable. Aspects of genetic variation are fixed throughout life, and increasingly straightforward to assay; most will likely be identified within the decade, after routine genome re-sequencing provides comprehensive catalogs of genome variants.

Investigation of the environmental influences on human behavioral phenotypes poses more fundamental questions. The environment encompasses a vast array of different components, some of which are distinct and objectively measurable – for example exposure to particular toxins – while others are generally poorly defined and their severity only assessed subjectively – such as stressful life events [1], [2]. The size and diversity of the environmental variable space make it difficult to select a manageable number of such variables for investigation in relation to behavioral phenotypes. Furthermore, the environment shifts throughout life, as does the impact of specific environmental variables. These complexities suggest the importance of establishing a framework for investigating environmental influences on behavior that fulfills three criteria: 1) enables consideration of a wide range of variables; 2) permits the evaluation of such variables longitudinally; and 3) allows for the joint analysis of these variables with genetic variation datasets, which are adequately powered to detect gene-environment interactions.

Longitudinal birth-cohorts uniquely provide such a framework. They offer the opportunity to assess the influence of multiple early environmental factors on the development of neurobehavioral profiles. Such cohorts also enable examination of the relationship between these profiles and overt expression of psychiatric illness and adult temperament while avoiding problems associated with sampling and recall bias. The Northern Finland 1966 Birth Cohort (NFBC 1966) is well suited to address these types of questions, as more than 10,000 individuals born in the year 1966 in the two most northern provinces of Finland have been followed over the course of their life, starting from before birth, until age 31. The NFBC 1966 database permits longitudinal analyses of sociodemographic characteristics, neurodevelopment, and quantitative neurobehavioral measures [3], in a large, relatively genetically homogeneous population.

Another example is the Dunedin Multidisciplinary Health and Development Study, from which a number of early childhood factors have been identified that predict the risk of developing post-traumatic stress disorder [4]. More generally, a review of similar longitudinal cohorts reveals that parental psychopathology, negative life events and prenatal stress, maternal smoking, in addition to low maternal age and education, have been shown to predict later psychopathology in children and adolescents [5], [6], [7], [8], and that socioeconomic status is be a moderator between early risk factors and externalizing and internalizing behavior in children [9]. A large body of literature thus supports the role of early environmental factors in influencing the development of psychopathology.

Temperament is considered a candidate endophenotype for a wide range of psychiatric disorders, reflecting common genetic factors shared across diagnostic categories [10]. As temperament develops early and remains moderately stable throughout life [11], it is not surprising that dimensions of temperament predict psychopathology in adulthood [12]. For example, high negative emotionality consistently predicts high levels of both externalizing and internalizing problems [11], [13]. Substantial evidence indicates that specific temperament dimensions predispose to psychopathology. Yet recent studies have suggested that *clusters* comprised of multiple temperament dimensions capture more information about individual differences and risk profiles [11], [14], [15], [16]. In particular, in the first of a pair of analyses that we report here, we conducted a cluster analysis of temperament in the NFBC1966 and demonstrate that adult temperament clusters predict adult psychiatric and somatic health better than individual dimensions of temperament alone (Wessman et al., *in press*).

In the second of this pair of analyses (presented here), we set out to further examine whether the temperament patterns seen in adulthood are consistent across the developmental trajectory. Specifically, we examined the relationship between prospective measures capturing the early environment, neurobehavioral development, and adolescent behavior (obtained from the extensive life course data available in NFBC 1966) and temperament clusters assessed in adulthood. Temperament, in our series of analyses, therefore represents a critical phenotype for examining the development of individual differences associated with adult health outcome. By conducting a data-driven investigation to uncover relationships between life course measures and adult temperamental profiles in this rich, longitudinal birth cohort, our approach is in contrast to many analyses of early environmental influences on temperament in longitudinal birth cohorts. First, conducting an exploratory analysis with a range of life course variables enabled us to comprehensively examine all variables, in order to identify suitable targets for future research, rather than limit our focus to a single known predictor. Second, by comparing the relationship between these life course variables and temperament profiles to the relationship between these variables and individual temperament scales, we were able to compare these different (i.e., person-oriented vs. variable-oriented) approaches to representing temperament. Although our data-driven approach did not involve testing a series of hypotheses about each prospective measure, we did hypothesize that:

1. We would identify early life course measures that could predict temperament clusters, just as we identified health and outcome correlates of temperament in adulthood in Wessman et al. (*in press*); and
2. We would identify associations between early life course measures and adult temperament clusters that are consistent with previous findings of risk factors for the development of psychopathology.

We note that the analysis conducted here does not allow us to make conclusions about causality (which is difficult to establish with life course measures and temperament). The analysis does, however, identify specific measures that are associated with the development of temperament features.

## Materials and Methods

### Participants

The Northern Finland 1966 Birth Cohort (NFBC 1966) is a longitudinal birth cohort, initially comprised of all 12,058 individuals live-born in 1966 from the two northernmost provinces of Finland, Oulu and Lapland [17]. The cohort members have been monitored prospectively from the prenatal period onwards. In particular, data on the cohort members’ socioeconomic status and family characteristics, health conditions, developmental milestones, education and behavior were collected prospectively from pregnancy up to age 31 years.

When cohort members were 31-years old, all subjects who were alive at the time and had a known address were asked to complete a subset (107 items) of Cloninger’s Temperament and Character Inventory (TCI) version 9 questionnaire for measurement of four dimensions of temperament (Novelty Seeking: NS, Harm Avoidance: HA, Reward Dependence: RD, and Persistence: P) and their respective subscales [18], [19]. NS is a tendency to respond with intense excitement to novel stimuli, or cues for potential rewards or potential relief of punishment and thereby activating behavior. HA is a tendency to respond intensively to signals of aversive stimuli, thereby inhibiting behavior. RD is a tendency to respond intensely to signals of reward, especially social rewards, thereby maintaining and continuing particular behaviors. P is a tendency to persevere in behaviors that have been associated with reward or relief from punishment. There are 12 subscales comprising these four dimensions: (HA1: anticipatory worry, HA2: fear of uncertainty, HA3: shyness, HA4: fatigability; NS1: exploratory excitability, NS2: impulsiveness, NS3: extravagance, NS4: disorderliness; RD1: sentimentality, RD3: attachment, RD4: dependence). Reference of collection and application of these scales is available [20], [21]. It has previously been shown that scores measured by the TCI distribute normally in the population with sex-dependent differences [22].

The current study sample contains cohort members who were living in Finland at the age of 16 years, who completed the TCI at the age of 31, who were not mentally retarded, and who provided informed consent (N = 3,761∶ 1,726 male, 2,035 female). All subjects included in the present study gave written consent for their data to be used. The study was approved by the Ethics Committee of the Faculty of Medicine, University of Oulu.

### Life Course Measures

As this was an exploratory analysis to identify any life course measure associated with adult temperament, we did not restrict our choice of variables based on *a priori* hypotheses. Rather, we took advantage of the multitude of life course variables available in the NFBC 1966 database and selected those for analysis of association with temperament at age 31 if sufficient information was available about the nature of the variable (i.e., how information was collected and measured) and more than 50% of cohort members had data available for that variable [3]. All variables are categorical, except for mother’s age, weight and ponderal index at birth, weight, height, and ponderal index at one year of age, adolescent weight and height, and average grades for all subjects in adolescence. For categorical variables, the possible categories are listed next to each variable in Tables S1, S2, S3, S4, S5. These 54 variables can be grouped into four general categories, which are described below and listed in Tables 1, 2, 3, 4, as well as Tables S1, S2, S3, S4, S5.

**Table 1:** Table of relationships between pre-natal sociodemograhic measures and group membership in temperament clusters or individual scales.

Predictors	Cluster I	Cluster II	Cluster III	Cluster IV	Scales_F	Scales_M
Primary parent occupation at birth			M			
Maternal education	F, M	M	F			P
Mother’s age		F, M	M		NS	HA
Mother lived in same region entire life	F				RD	RD
Ratio children/household rooms						
Home location at birth	F				NS	
Distance to maternity clinic						
Distance to neighbors						HA
Distance to city/town center						RD
Distance to doctor	F				HA	RD
Household has running electricity					RD	
Household has running water		F				
Household has running car						
Family owns home at birth				F	HA	
Mother worked outside of home during pregnancy			M		HA	
How strenuously the mother worked during the pregnancy						
Mother exposed to outside information during pregnancy		M			NS	HA
Desirability of the pregnancy			M	M		
Mother’s frame of mind during the pregnancy	F					
Mother smoked during the pregnancy						
Maternity clinic visits						

Significant predictors are indicated by an F for female or M for male Cluster I–IV membership or the scale name for individual TCI scales. Scales_F: predictors of individual TCI scales for females; Scales_M: predictors of individual TCI scales for males.

**Table 2:** Table of relationships between infant developmental milestones and group membership in temperament clusters or individual scales.

Predictors	Cluster I	Cluster II	Cluster III	Cluster IV	Scales_F	Scales_M
Birth weight						
Birth height					P	
Ponderal index at birth						
Weight at one year						
Height at one year						
Ponderal index at one year						
Age of standing						
Age of walking without support						
Number of words spoken by age one		F			HA, NS	RD
Child wets self during the day at age one					HA	HA, RD
Child wets self during the night at age one					NS	
Potty-training age one						

Significant predictors are indicated by an F for female or M for male Cluster I–IV membership or the scale name for individual TCI scales. Scales_F: predictors of individual TCI scales for females; Scales_M: predictors of individual TCI scales for males.

**Table 3:** Table of relationships between family and health characteristic through adolescence and group membership in temperament clusters or individual scales.

Predictors	Cluster I	Cluster II	Cluster III	Cluster IV	Scales_F	Scales_M
Father’s occupation in adolescence				F	HA, NS	NS
Family status						
Home location in adolescence				F, M	NS	
Weight in adolescence						
Height in adolescence			F	F		
Number of hospital visits from 1966–1987						
Number of long-duration illnesses.						

Significant predictors are indicated by an F for female or M for male Cluster I–IV membership or the scale name for individual TCI scales. Scales_F: predictors of individual TCI scales for females; Scales_M: predictors of individual TCI scales for males.

**Table 4:** Table of relationships between educational milestones and behavior through adolescence and either group membership in temperament clusters or individual scales.

Predictors	Cluster I	Cluster II	Cluster III	Cluster IV	Scales_F	Scales_M
School level classification				M		HA
Average grades in adolescence	F, M		F, M	F	P	
Repeated grade in school						RD
Admitted to secondary school	F				RD	RD
School admission				F		
Times applied to secondary school	M		M	M		RD
Physical education grades in adolescence		F	M	M	HA	
Frequency of sports outside of school				F	HA	HA
Smoking in adolescence	F	F	F	F	NS	
Drinking in adolescence					HA, NS	
Being drunk in adolescence		M				NS
Intoxicant use in adolescence						HA, NS

Significant predictors are indicated by an F for female or M for male Cluster I–IV membership or the scale name for individual TCI scales. Scales_F: predictors of individual TCI scales for females; Scales_M: predictors of individual TCI scales for males.

#### Pre-natal sociodemographic environment

The following sociodemographic characteristics were selected from a questionnaire completed in the 24^th^ to 28^th^ gestational week (Table 1): family socioeconomic status in 1966, based on the occupation of the primary parent (categorized into skilled vs. unskilled professions); mother’s education; mother’s age; whether the mother had always lived in the same village, town, or city; the ratio of number of children to number of rooms in the household; location of the home (city, small town, rural center, or remote village); distance of the home to key resources, including maternity clinic, neighbors, city/town center, and doctor’s office; availability of electricity, running water, and car; and whether the family owned their own home or not. The following characteristics about the mother’s health and the pregnancy were selected from the same questionnaire: how often, and how strenuously, the mother worked during the pregnancy; whether the mother was exposed to outside information about lifestyle and health; whether the pregnancy was wanted; the mother’s frame of mind during the pregnancy (i.e., depressive symptoms); whether the mother smoked during the pregnancy; and the number of visits to the maternity clinic.

#### Infant developmental milestones

A selection of variables representing infant developmental milestones and health were selected from data collected during the cohort members’ examination by nurses performed at one year of age (Table 2). In addition to birth weight, height, and ponderal index, the following characteristics of the infant’s development were selected: weight, height, and ponderal index at one year; age of standing and walking without support; number of words spoken; whether day/night time wetting occurred; and whether potty-training had occurred.

#### Family and health characteristics through adolescence

A selection of variables reflecting family characteristics and adolescent health were selected from a questionnaire mailed to the cohort members at age 14, in addition to information obtained from the national health registry (Table 3). The following sociodemographic characteristics were selected: family socioeconomic status in 1980, based on the father’s occupation (categorized into skilled vs. unskilled professions); family status (both parents present, one parent present, both parents deceased); and location of the home (urban vs. rural). The following health-related characteristics were selected: weight and height; the number of hospital visits from 1966–1987; and the number of long-duration illnesses.

#### Educational milestones and behavior through adolescence

Educational attainment and adolescent behavior characteristics were selected from a questionnaire mailed to the cohort members in 1980, in addition to information obtained from the Joint Application System (which is a nationwide application system through which cohort members applied to secondary level education) in 1982 (Table 4). The following measures were selected: classification of school level at age 14 (above or below the median); average grades for all subjects; the number of times a grade was repeated in school; whether they were admitted to secondary level education; what type of school they were admitted to (secondary, vocational, both, neither, or didn’t apply); and the number of times they applied to secondary level education. Lastly, the following characteristics of adolescents’ behavior were selected: average grade for physical education; the frequency of sports activity outside of school; and self-reported rates of smoking, drinking, drunkenness, and intoxicant use.

### Data Analysis

#### Temperament clusters

Cluster analysis, using the *k*-means method, of TCI scores was previously conducted, and the characterization of the clusters (Clusters I–IV) is reported in Wessman et al. (*in press*). Briefly, k-means clustering was conducted on the 12 TCI subscale scores, separately for each sex (as the distribution of the subscales in the two genders differ significantly [19], [22]), in the total sample of 3,761 individuals; the algorithm was computed with 2–12 clusters selected, and the best model was selected based on the Bayesian information criterion [23]. Results of these cluster analyses, which are detailed in our parallel manuscript, revealed an optimum of four clusters. These four stable and robust clusters were consistent between sexes, and the stability of this structure was further supported by a replication analysis in a separate population sample of >2,000 Finnish individuals (Wessman et al., *in press*).

The resulting clusters obtained from these prior analyses are described briefly here (and in greater detail in our concurrent manuscript, Wessman et al., *in press*), and apply to both males and females. For females, Clusters I, II, III and IV include 26%, 25%, 28% and 21% of the subjects, whereas for males these numbers are 26%, 22%, 30% and 22%. Cluster I individuals are characterized by high persistence (P), low extravagance (NS3), anticipatory worry (HA1) and fatigability (HA4). Cluster II individuals are characterized by very low fear of uncertainty (HA2), shyness (HA3) and very high exploratory excitability (NS1), impulsiveness (NS2), extravagance (NS3), disorderliness (NS4), as well as above average persistence (P) and attachment (RD3). Although Cluster III individuals show relatively average temperament scores, these individuals are characterized by low persistence (P) but high dependence (RD4) and extravagance (NS3). Cluster IV individuals are characterized by particularly high levels of high anticipatory worry (HA1), fear of uncertainty (HA2), shyness (HA3), fatigability (HA4), paired with low exploratory excitability (NS1) and attachment (RD3). As such, individuals from Cluster I can be described as stable, persistent and not very impulsive; from Cluster II as outgoing, energetic and impulsive; from Cluster III as not extreme on any trait dimension; and from Cluster IV as shy, pessimistic, and with a preference for routine and privacy.

#### Multivariate association analyses

In the present analyses, we attempted, using a multivariate analysis, to identify life course measures that were significantly associated with membership in the above four clusters (I–IV). To reduce the number of variables for consideration in the multivariate models, we first conducted a series of univariate analyses, separately by sex, in order to examine differences between temperament clusters in early life variables. These univariate analyses consisted of one-way analyses of variance for continuous variables and chi-square tests for categorical variables, and were conducted using R statistical software (R 2.9.2) (http://www.r-project.org). The p-values for contingency table analyses of categorical variables were determined using an MCMC approximation to Fisher’s Exact Test (1,000,000 replicates). A total of 54 independent early life variables were tested for differences between the 4 clusters, separately for both sexes.

After initial univariate analyses, we conducted four stepwise logistic regression analyses for each sex. The outcome in these logistic regression analyses was an indicator variable for membership in one of the four clusters. By entering all variables that significantly predicted cluster differences in univariate analyses (at a *p*<0.05 uncorrected level), we identified the set of variables that jointly predicted group membership in each of the clusters, and for each sex, separately, while controlling for all significant predictor variables. Models with the lowest AIC were chosen as the final model.

#### Temperament dimensions

To examine life course measures in relation to temperament dimensions, we followed a similar analysis plan to that employed for the temperament clusters, specifically using univariate analyses to identify candidate independent life course variables followed by a multivariate analysis. The difference between these analyses was that for each of the TCI scales (NS, HA, RD, and P), and for each sex, we used linear models (rather than logistic models used for temperament clusters) to predict the temperament values as a function of each life course variable.

After initial univariate analyses, we conducted four stepwise linear regression analyses. By entering all variables that significantly predicted dimension scores in univariate analyses (at a *p*<0.05 uncorrected level), we identified the set of variables that predict temperament dimension scores while controlling for all significant predictor variables, for each sex separately. Models with the lowest AIC were chosen as the final model.

#### Temperament clusters vs. dimensions

To examine the relative correlation of life course measures with temperament cluster membership as compared to temperament dimensions, we present a generalized r^2^ for the logistic models [24] and the coefficient of determination for the linear models. Both measures attempt to measure the amount of variability in the outcome (cluster membership or temperament dimension) that is captured by the suite of life course variables retained in the final multivariate models.

## Results

### Individuals that Differ from Each Other Based on Adult Temperament Show Significant Differences in a Number of Prospective Life Course Measures

Our univariate analyses reveal multiple variables that significantly differed between clusters (Tables S1, S2, S3) and predicted scale scores (Tables S4–S5). As the purpose of these univariate analyses was only to identify variables to be used in multivariate analyses, they will not be discussed further.

### A Suite of Life Course Measures Predict Adult Temperament Clusters

Tables 1, 2, 3, 4 reflect the significant predictors of each cluster and for each individual scale, for males and females separately, and are grouped by the four general categories of life course measures. Tables S6, S7, S8, S9 present the regression coefficients and *p*-values for each significant predictor, and are grouped separately by gender, as well as by the four clusters (Tables S6–S7) and individual TCI scales (Tables S8–S9).

Stepwise logistic regression analyses revealed sets of variables that significantly predict group membership for each cluster and each sex separately (Tables 1, 2, 3, 4; Tables S6). For example, predictors of Cluster II membership for females included mother’s age, whether the household at birth had running water, the number of words spoken by the cohort member at age one (particularly whether the child spoke three or more words by age one), physical education grades in adolescence, and smoking in adolescence. In particular, the odds of being in Cluster II are approximately 2 times as great for female adolescents who reported smoking occasionally (OR = 2.30, *p*<0.0005) or twice a week or more (OR = 2.42, *p*<0.0005), as compared to female adolescents who reported never smoking in adolescence (Tables 1, 2, 3, 4; Table S6).

A comparison across clusters reveals that a relatively consistent set of life course measures predicts group membership, including maternal education, characteristics of the family’s location and residence, adolescent academic performance, and adolescent smoking. In particular, the odds of being in Cluster I for both males and females decreases, while the odds of being in Cluster II for males and Cluster III for females increases, with increasing maternal education. In terms of the prenatal sociodemographic environment, for females the odds of Cluster I membership decrease as households become more rural and distant from key resources, the odds of Cluster II membership decrease as families report not having running water, and the odds of Cluster IV membership decrease as families report not owning their own home. In terms of educational milestones and behavior at age 14, the odds of being in Cluster I (females and males) increase, while the odds of being in Cluster III (females and males) and IV (females) decrease, with increasing grades. The odds of being in Cluster II increase with increasing physical education grades for females, while the odds of being in Cluster IV decreases with increasing physical education grades for males. Finally, with increasing smoking reported by females in adolescence, the odds of either Cluster II or III membership increases, but the odds of either Cluster I or IV membership decreases. The presentation of Tables 1, 2, 3, 4 is designed to highlight those sets of variables that are significantly associated across clusters.

### A Consistent Suite of Life Course Measures Predict Cluster Membership while there is Less Consistency in the Measures that Predict Individual Temperament Dimensions

A comparison of generalized r^2^ values from the logistic models (Tables S6–S7) and coefficients of determination from the linear models (Tables S8–S9) indicates that all models account for less than 10% of the variation in outcome classifications (cluster membership or temperament dimension). Examination of the variables retained in the final multivariate models reveals that the same measures are included in the final models for more than one cluster while most measures are unique to the final models for each temperament scale. For example, maternal education is retained in the final model for Clusters I and III (females) and Clusters I and II (males). In contrast, maternal education is only retained in the final model of Persistence (males).

Furthermore, in terms of the measures that significantly predict temperament scale scores, there is little consistency of variables across scales or across sexes. The only measures that consistently predict scale scores for both sexes are mother’s lifetime residence for RD, whether the child wets him/herself during the day at age one for HA, and sports frequency in adolescence for HA. In contrast to the suite of life course measures that predict more than one cluster, there are no shared variables that significantly predict scores across temperament scales. This contrast is highlighted in Tables 1, 2, 3, 4, where a consistent pattern is evident across clusters, but not dimensions.

## Discussion

This is the first report to demonstrate that life course measures (assessed as early as before birth) significantly predict adult temperament (assessed at age 31). Although some prior evidence has suggested specific developmental pathways with implications for psychopathology leading from early environment to adult temperament, such evidence derives from studies using limited age ranges or retrospective data. In the series of analyses reported here, we first observed that stable and robust clusters of temperament differ on a number of variables that were assessed at age 31 in the NFBC 1966, including lifestyle, working capacity, socioeconomics status, and mental health (Wessman et al., *in press*).

The goal of these analyses was to identify sociodemographic, developmental, and behavioral correlates, as measured prenatally, in infancy and into adolescence, of adult outcome as indicated by temperament profiles. By conducting a data-driven investigation using a longitudinal birth-cohort, we are able to demonstrate that a set of life course measures predict adult temperament clusters, revealing both novel relationships and confirming similarly reported associations. Although we do not make any claims about causation, based on our findings we propose that these clusters represent distinct temperament profiles and that information about a subset of life course measures has implications for adult health outcomes.

### Individuals that Differ from Each Other Based on Adult Temperament Show Significant Differences in a Number of Prospective Life Course Measures

These findings have implications for our understanding of the development of individual differences in temperament, as well as mental health outcome in adulthood. It has been shown that specific early environmental risk factors influence psychiatric susceptibility [5], [6], [7], [8], and it has also been shown that dimensions of childhood temperament predict psychopathology [11], [12], [13]. However, most of these studies are initiated after birth and often are limited to childhood or adolescence only.

In support of our first hypothesis – that we would identify early life course measures that predict adult temperament clusters – we were able to demonstrate that life course measures assessed as early as the prenatal period are associated with membership in distinct clusters organized according to temperament in adulthood, and that these differences seen across the life course are consistent with differences between clusters seen in habits, socioeconomic status, and health in adulthood (Wessman et al., *in press*). Although it has previously been demonstrated that children and adolescents characterized by differences in temperament [14], [15], [25], problem behavior [26], or both [27] follow distinct developmental trajectories, this is the first study to elucidate individual differences over the life course of a longitudinal cohort using *prospectively* assessed measures.

### A Suite of Life Course Measures Predict Adult Temperament Clusters

The results of our multivariate analyses suggest that a relatively consistent set of life course measures are associated with adult temperament profiles, including maternal education, characteristics of the family’s location and residence, adolescent academic performance, and adolescent smoking. In support of our second hypothesis – that we would identify associations between early life course measures and adult temperament that are consistent with previous risk factors for the development of psychopathology – the set of life course measures identified in our analyses are in line with previous reports. For example, maternal education has been associated with children’s problem behavior, such that increasing maternal education protects against the development of problem behaviors at ages 2 and 5 [6]. Maternal education has also been associated with adolescent temperament, such that less education has been associated with the adolescent offspring having low perceptions of self-worth and academic competence, whereas more maternal education has been associated with the adolescent offspring having moderately high self-regulation, low risk proneness, and moderately high perceptions of self-worth and academic competence [14]. Here, we demonstrate that less maternal education is associated with temperament profiles characterized by low NS and HA, but high P (Cluster I), whereas more maternal education is associated with temperament profiles characterized by low HA but high NS in females (Cluster II) and average temperament scores in males (Cluster III). Although not consistent across sexes, this set of observations suggests a relationship between maternal education and the combination of NS and HA within offspring.

Adolescent smoking has also been implicated as playing an important role in the developmental trajectory as it is predicted by early life measures (particularly family socioeconomic status) [7]. It has been shown to discriminate among clusters of adolescents characterized by problem behaviors and to be associated with an adolescent temperament profile that is rigid and distractible, active, not persistent, and characterized by poor mood [27]. Here, we demonstrate that adolescent self-reported levels of smoking discriminate between female clusters, as low levels of smoking in adolescence is associated with a combination of low HA and NS (Clusters I and IV), whereas high levels of smoking is associated with moderate-to-high HA and NS scores (Clusters II and III).

The comprehensive assessment of life course measures in this cohort therefore allows for the elucidation of a set of correlates that potentially play an important role in the development of individual differences in temperament. The set of variables that consistently predicts adult temperament across the four clusters reflects the growth environment (such as maternal characteristics or the nature of the home environment) or the early, emerging temperament of cohort members (such as academic performance). The variables related to the growth environment may reflect the background of emerging temperament. Alternatively, as the development of temperament is under genetic control, these variables may interact via mechanisms of genetic correlation, as the genetic background of the parents (with whom the offspring shares genes) affects the growth environment.

### A Consistent Suite of Life Course Measures Predict Cluster Membership while there is Less Consistency in the Measures that Predict Individual Temperament Dimensions

Overall our findings suggest that a suite of life course measures predicts membership across temperament clusters. While these measures may be either shared between temperament clusters (maternal education) or unique to a given cluster (number of words spoken at age one), the measures that predict temperament dimensions are unique to particular temperament scales. In the accompanying report, we demonstrate that these temperament clusters are significantly related to adult outcome across a number of lifestyle and health domains and that the proportion of variables significantly associated with clusters is similar to the proportion of variables significantly associated with any subscale, suggesting that these clusters capture as much information about adult outcome as individual scale scores alone (Wessman et al., *in press*). We argue here that these temperament clusters capture more information about the development of temperament profiles than the scales alone.

One advantage of organizing adult temperament according to such clusters is that this strategy reduces the number of variables to be tested. An additional advantage is that it provides the opportunity to consider the context of the individual’s temperamental profile and environmental influences, so that it is possible to consider how different combinations of temperament dimensions assort within individuals, rather than requiring the assumption that dimensions operate independently [15], [16]. The use of such a clustering approach is supported by our findings that a shared set of variables predicts membership across clusters, whereas only non-overlapping sets of variables predict temperament dimensions.

### Relationship between Results Presented here and Results Presented by Wessman et al. (In press)

In our first set of analyses (Wessman et al., *in press*) we conducted a cluster analysis of temperament sub-scales using the NFBC 1966, which provided evidence in favor of four stable and robust clusters of temperament, which were similar between genders and which we labeled Clusters I–IV. We next examined the association between these temperament clusters and a broad range of measures of health and well being that were assessed in adulthood. Our results demonstrate clear patterns of association between temperament clusters and health, life events, and well-being: Cluster I individuals are characterized by healthy life habits, stable life features, and a decreased risk for mental illness; Cluster II individuals report high physical fitness, education and annual income, higher smoking and alcohol use, in addition to high scores on a hypomania personality scale; Cluster III individuals are not characterized by extreme characteristics in lifestyle or health; and Cluster IV individuals are characterized by the lowest scores in most areas of health and well-being, and are at increased risk for physical and mental illness. In summary, the analyses in Wessman et al. (*in press*) characterized the health and well-being profiles of these four temperament clusters, using data collected only in adulthood.

In the analyses reported here, we extended our analyses longitudinally, and assessed the relationship between the temperament clusters and a broad range of sociodemographic, developmental, and behavioral measures that were measured prenatally, in infancy and into adolescence. Our results suggest that a relatively consistent set of life course measures are associated with adult temperament profiles, including maternal education, characteristics of the family’s location and residence, adolescent academic performance, and adolescent smoking.

Considering these sets of findings together, our results provide additional support for such a person-oriented approach, and increase our understanding of the factors that contribute to the trajectory of individual differences in temperament, which in turn influence adult mental and physical health outcomes.

### Strengths and Limitations

The primary strength of this report is the use of a longitudinal birth cohort that allowed us to investigate whether sociodemographic, developmental, and behavioral variables that were assessed prenatally and through development predict temperament scores assessed in adulthood. Our analyses of the NFBC 1966 allowed us to examine the influence of multiple life course measures on temperamental profiles while avoiding problems associated with sampling and recall bias. Our approach to analyzing all 54 life course measures in relation to temperament was exploratory: each variable was treated the same (e.g., not ordered), considered independently at the first stage of univariate analyses, and carried forward to the second stage of multivariate analyses if significant. Although there is potentially some overlap in some of the items, we chose to analyze all variables that were available, if they were sufficiently described and if more than 50% of cohort members had data available for that variable, in order to examine as much as the environmental search space as possible.

The primary limitation of this report is that the information available for analysis is constrained by what was collected in the cohort. For example, we did not have a direct index of socioeconomic status available for the families of cohort members, nor did we have a measure of fetal alcohol exposure. In addition, despite the rich dataset collected on this cohort, temperament was only assessed in adulthood. Future work should be aimed at repeated measurement of temperament, as well as socioeconomic status and family characteristics, health conditions, developmental milestones, education and behavior, across the life course.

We also did not have equal sample sizes for males and females, which complicates interpretation of differences in results across sexes. The NFBC 1966 began with a cohort of 12,058 live births; here, our analyses were conducted on a total of approximately 1,400 of those individuals. While it has previously been demonstrated that study participation is lower in individuals with a psychiatric illness as compared to those without, participation does not vary across specific disorders [21]. However, we cannot rule out possible effects of selective attrition on our results. It is possible that the resulting four-cluster structure, or the association between these temperament clusters and life course variables, would differ if the entire cohort were available for analysis. Furthermore, life course measures reflect both genetic and environmental influences on the developmental trajectories and we cannot make conclusions about causation. However, by taking an exploratory approach, we are able to identify life course candidates that are potentially causative, which provide suitable targets for future investigation.

Finally, as we have already stated, our analysis does not allow us to make conclusions about causality, but identifies specific measures that are associated with the development of temperament features. In addition, it is also possible that associations reported here are indirect, such that an additional, unmeasured variable is responsible for their association. Additional comprehensive and longitudinal cohorts will be critical to uncovering the mechanisms underlying temperament and the development of psychopathology.

### Conclusion

Early environment, neurobehavioral development, and adolescent behavior significantly predict adult temperament. Although all multivariate models account for less than 10% of the variation in outcome classifications (both cluster membership and temperament dimension), our results highlight a consistent set of life course measures that predict temperament clusters. Of note, we were able to replicate previous associations between early life variables (e.g., maternal education) and adult temperament, even when considering a large set of life course measures. These results contribute to our understanding of how individual differences in life course correlates are related to individual differences in adult temperament, and support the utility of conducting data-driven research to both uncover novel, and replicate previously reported, associations.

Our results demonstrate significant relationships between life course measures and temperament clusters, particularly in females. There is substantial evidence that risk factors for later psychopathology include parental psychopathology, low socioeconomic status, prenatal stress and the experience of negative life events, maternal smoking, a low maternal age and education [5], [6], [7], [8], [9], [28], [29], [30]. In particular, it is clear that negative life events experienced by the family, particularly the mother, predict the development of early problem behaviors and psychopathology, directly and independently of family structure, socioeconomic status, or maternal psychopathology [6], [8], [28], [30]. Our findings that life course measures significantly differ between different temperament clusters in adulthood suggests that the influence of early environment is not limited to psychopathology, but also extends to the development of stable and robust temperament dimensions.

## Supporting Information

Table S1 **Differences in early life measures between female temperament clusters.**
(DOC)

Table S2 **Differences in early life measures between male temperament clusters.**
(DOC)

Table S3 **Differences in average grades in adolescence between temperament clusters for females and males.**
(DOC)

Table S4 **Early life measures predicting individual temperament dimensions, as measured by the Temperament and Character Inventory, which survived correction for females.**
(DOC)

Table S5 **Early life measures predicting individual temperament dimensions, as measured by the Temperament and Character Inventory, which survived correction for males.**
(DOC)

Table S6 **Early life measures predicting group membership of each female temperament cluster separately.**
(DOC)

Table S7 **Early life measures predicting group membership of each male temperament cluster separately.**
(DOC)

Table S8 **Early life measures predicting temperament dimension scores for females.**
(DOC)

Table S9 **Early life measures predicting temperament dimension scores for males.**
(DOC)
