# Supplementary material for: Early Environment and Neurobehavioral Development Predict Adult Temperament Clusters
Source: PLoS One. 2012 Jul 18;7(7):e38065. doi: 10.1371/journal.pone.0038065 (PMC3399831; doi:10.1371/journal.pone.0038065)
Supplement: Table S1 — Differences in early life measures between female temperament clusters. (DOC) [file pone.0038065.s001.doc]

**Table S1. Differences in early life measures between female temperament clusters.**

|  |  | Temperament Clusters | | | |
| --- | --- | --- | --- | --- | --- |
|  |  | I | II | III | IV |
| *Prenatal Sociodemographic Environment* | | | | | |
| Primary Parent Occupation | Unskilled | 25.33% | 20.00% | 19.65% | 31.41% |
|  | Skilled | 74.67% | 80.00% | 80.35% | 68.59% |
|  | χ^2^ (*N* = 2,035) = 23.91, *p* = 2.3 x 10^-5^, *V* = 0.11 | | | | |
| Maternal Education | No or 1-4 years primary school | 11.93% | 8.52% | 7.54% | 15.06% |
|  | 5-8 years or unfinished primary school | 58.90% | 57.61% | 54.94% | 59.53% |
|  | Some or over 2 years vocational school | 16.29% | 16.43% | 21.54% | 17.65% |
|  | 5 or more years secondary school | 10.23% | 12.17% | 12.39% | 6.35% |
|  | Matriculation or more | 2.65% | 5.27% | 3.59% | 1.41% |
|  | χ^2^ (*N* = 2,003) = 43.87, *p* = 1.8 x 10^-5^, *V* = 0.09 | | | | |
| Home Location | City | 30.91% | 35.40% | 33.10% | 18.48% |
|  | Small town | 3.35% | 2.40% | 1.59% | 1.85% |
|  | Rural center | 23.84% | 26.20% | 25.84% | 23.33% |
|  | Remote village | 41.90% | 36.00% | 39.47% | 56.35% |
|  | χ^2^ (*N* = 2,035) = 56.94, *p* = 1.0 x 10^-6^, *V* = 0.10 | | | | |
| Distance to maternity clinic | Less than 300 m | 10.88% | 10.12% | 8.51% | 5.46% |
|  | 300 m – 2.9 km | 37.52% | 44.33% | 42.21% | 29.22% |
|  | 3 – 9.9 km | 19.32% | 18.22% | 19.20% | 24.47% |
|  | 10 – 16.9 km | 12.95% | 12.55% | 14.49% | 17.34% |
|  | 17 – 23.9 km | 7.69% | 5.06% | 4.71% | 6.89% |
|  | 24 – 30.9 km | 6.00% | 4.05% | 3.44% | 6.41% |
|  | 31 – 100 km or more | 5.63% | 5.67% | 7.43% | 10.21% |
|  | χ^2^ (*N* = 2,000) = 31.74, *p* = 1.06 x 10^-6^, *V* = 0.07 | | | | |
| Distance to town center | Less than 300 m | 46.39% | 52.57% | 48.09% | 35.61% |
|  | 300 m – 2.9 km | 7.60% | 7.60% | 6.56% | 4.95% |
|  | 3 – 9.9 km | 11.03% | 9.24% | 11.29% | 13.68% |
|  | 10 – 16.9 km | 9.89% | 10.27% | 10.75% | 13.68% |
|  | 17 – 23.9 km | 8.17% | 3.70% | 4.01% | 5.66% |
|  | 24 – 30.9 km | 5.51% | 4.11% | 3.83% | 6.84% |
|  | 31 – 100 km or more | 11.41% | 12.53% | 15.48% | 19.58% |
|  | χ^2^ (*N* = 1,986) = 31.66, *p* = 2.2 x 10^-16^, *V* = 0.07 | | | | |
| Distance to doctor | Less than 300 m | 9.51% | 9.50% | 7.66% | 3.80% |
|  | 300 m – 2.9 km | 30.42% | 34.50% | 31.93% | 24.23% |
|  | 3 – 9.9 km | 16.73% | 18.39% | 19.71% | 19.48% |
|  | 10 – 16.9 km | 12.93% | 11.78% | 12.04% | 14.96% |
|  | 17 – 23.9 km | 9.13% | 6.20% | 5.84% | 6.18% |
|  | 24 – 30.9 km | 7.22% | 5.79% | 6.20% | 8.79% |
|  | 31 – 100 km or more | 14.07% | 13.84% | 16.61% | 22.57% |
|  | χ^2^ (*N* = 1,979) = 30.54, *p* = 1.0 x 10^-6^, *V* = 0.07 | | | | |
| Household has electricity | Yes | 86.39% | 88.31% | 86.89% | 78.17% |
|  | No | 13.61% | 11.69% | 13.11% | 21.83% |
|  | χ^2^ (*N* = 2,008) = 22.43, *p* = 5.7 x 10^-5^, *V* = 0.11 | | | | |
| Own home | Yes | 53.40% | 44.83% | 46.95% | 61.63% |
|  | No | 46.60% | 55.17% | 53.05% | 38.37% |
|  | χ^2^ (*N* = 1,957) = 30.91, *p* = 3.0 x 10^-6^, *V* = 0.13 | | | | |
| *Infant Developmental Milestones* | | | | | |
| Potty-trained (How often the child defecate into a potty) | Never | 29.26% | 20.74% | 28.35% | 34.94% |
|  | Occasionally | 27.25% | 31.22% | 31.42% | 30.38% |
|  | Mostly | 26.45% | 31.88% | 28.93% | 23.54% |
|  | Always | 17.03% | 16.16% | 11.30% | 11.14% |
|  | (χ^2^ (*N* = 1,874) = 33.18, *p* = 0.0001, *V* = .08 | | | | |
| *Family and Health Characteristics through Adolescence* | | | | | |
| Home Location | Urban | 38.55% | 48.40% | 41.77% | 28.87% |
|  | Rural | 61.45% | 51.60% | 58.23% | 71.13% |
|  | χ^2^ (*N* = 2,035) = 38.30, *p* = 1.0 x 10^-6^, *V* = 0.14 | | | | |
| *Educational milestones and Behavior through Adolescence* | | | | | |
| Physical education grades | 7 or below | 23.33% | 19.54% | 26.78% | 32.51% |
|  | 8 | 45.51% | 43.70% | 47.19% | 46.40% |
|  | 9 | 26.96% | 31.09% | 23.22% | 19.35% |
|  | 10 | 4.21% | 5.67% | 2.81% | 1.74% |
|  | χ^2^ (*N* = 1,936) = 26.49, *p* = 7.0 x 10^-6^, *V* = 0.07 | | | | |
| Frequency of sports outside of school | Everyday | 15.05% | 13.84% | 11.28% | 8.27% |
|  | Every other day | 16.38% | 14.47% | 14.05% | 11.68% |
|  | Twice a week | 26.29% | 23.90% | 22.92% | 18.00% |
|  | Once a week | 18.48% | 20.75% | 20.15% | 25.06% |
|  | Every second week | 4.19% | 4.82% | 3.51% | 3.89% |
|  | Once a month | 3.43% | 3.98% | 5.36% | 3.65% |
|  | Usually never | 16.19% | 18.24% | 22.74% | 29.44% |
|  | χ^2^ (*N* = 1,954) = 38.74, *p* = 2.2 x 10^-16^, *V* = 0.08 | | | | |
| Smoking | Never tried | 40.98% | 30.17% | 29.65% | 41.06% |
|  | Tried once | 25.75% | 22.31% | 27.07% | 21.98% |
|  | Tried twice or more | 20.86% | 24.17% | 25.97% | 23.19% |
|  | Smoke occasionally | 6.39% | 12.81% | 10.13% | 8.94% |
|  | Smoke twice a week or more | 6.02% | 10.54% | 7.18% | 4.83% |
|  | χ^2^ (*N* = 1,973) = 47.29, *p* = 3.0 x 10^-6^, *V* = 0.09 | | | | |
| Alcohol use | Never drunk any | 46.15% | 33.75% | 36.46% | 46.38% |
|  | Tasted once | 32.65% | 32.71% | 35.91% | 34.30% |
|  | Drank few times, use alcohol monthly or weekly | 21.20% | 33.54% | 27.62% | 19.32% |
|  | χ^2^ (*N* = 1,973) = 39.65, *p* = 1.0 x 10^-6^, *V* = 0.10 | | | | |
| Being drunk | Never | 80.60% | 68.05% | 70.77% | 79.90% |
|  | Once slightly | 6.78% | 7.68% | 10.85% | 7.99% |
|  | Twice or more times slightly | 6.40% | 12.03% | 10.48% | 7.26% |
|  | Once very much | 1.88% | 4.56% | 3.31% | 2.42% |
|  | Several or more times very much | 4.33% | 7.68% | 4.60% | 2.42% |
|  | χ^2^ (*N* = 1,970) = 45.43, *p* = 8.0 x 10^-6^, *V* = 0.09 | | | | |

Note. A total of 54 independent variables were tested for differences between the 4 clusters, separately for both sexes. For females, Chi-square results and Cramer’s V coefficient for those variables that remained significant after Bonferroni correction (*p* < 0.00046) are presented.
