# Supplementary material for: Early Environment and Neurobehavioral Development Predict Adult Temperament Clusters
Source: PLoS One. 2012 Jul 18;7(7):e38065. doi: 10.1371/journal.pone.0038065 (PMC3399831; doi:10.1371/journal.pone.0038065)
Supplement: Table S2 — Differences in early life measures between male temperament clusters. (DOC) [file pone.0038065.s002.doc]

Table S2**.** Differences in early life measures between male temperament clusters.

|  |  | Temperament Clusters | | | |
| --- | --- | --- | --- | --- | --- |
|  |  | I | II | III | IV |
| *Prenatal Sociodemographic Environment* | | | | | |
| Primary Parent Occupation | Unskilled | 24.50% | 20.57% | 19.88% | 27.89% |
|  | Skilled | 75.50% | 79.43% | 80.12% | 72.11% |
| Maternal Education |  |  |  |  |  |
|  | No or 1-4 years primary school | 11.62% | 4.50% | 11.57% | 10.24% |
|  | 5-8 years or unfinished primary school | 55.13% | 56.61% | 55.49% | 61.99% |
|  | Some or over 2 years vocational school | 20.05% | 20.63% | 17.65% | 16.71% |
|  | 5 or more years secondary school | 7.97% | 14.02% | 10.59% | 8.63% |
|  | Matriculation or more | 5.24% | 4.23% | 4.71% | 2.43% |
| Home Location | City | 26.95% | 31.25% | 31.58% | 22.89% |
|  | Small town | 1.78% | 3.65% | 2.73% | 3.16% |
|  | Rural center | 28.73% | 27.60% | 25.34% | 24.74% |
|  | Remote village | 42.54% | 37.50% | 40.35% | 49.21% |
| Distance to maternity clinic | Less than 300 m | 10.16% | 12.40% | 14.03% | 8.51% |
|  | 300 m – 2.9 km | 39.28% | 39.58% | 38.93% | 37.23% |
|  | 3 – 9.9 km | 18.96% | 19.53% | 18.38% | 17.55% |
|  | 10 – 16.9 km | 13.32% | 14.78% | 12.06% | 15.69% |
|  | 17 – 23.9 km | 5.64% | 3.96% | 5.53% | 8.51% |
|  | 24 – 30.9 km | 4.51% | 5.01% | 4.74% | 6.12% |
|  | 31 – 100 km or more | 8.13% | 4.75% | 6.32% | 6.38% |
| Distance to town center | Less than 300 m | 48.64% | 54.64% | 50.91% | 45.95% |
|  | 300 m – 2.9 km | 5.45% | 5.31% | 4.23% | 4.05% |
|  | 3 – 9.9 km | 9.09% | 8.75% | 11.47% | 10.54% |
|  | 10 – 16.9 km | 11.59% | 10.08% | 10.26% | 10.54% |
|  | 17 – 23.9 km | 5.00% | 3.98% | 5.23% | 5.68% |
|  | 24 – 30.9 km | 5.45% | 6.10% | 5.43% | 6.76% |
|  | 31 – 100 km or more | 14.77% | 11.14% | 12.47% | 16.49% |
| Distance to doctor | Less than 300 m | 8.56% | 8.89% | 9.29% | 6.20% |
|  | 300 m – 2.9 km | 29.05% | 33.15% | 34.95% | 31.81% |
|  | 3 – 9.9 km | 16.67% | 16.98% | 14.95% | 15.36% |
|  | 10 – 16.9 km | 13.96% | 13.48% | 11.52% | 12.13% |
|  | 17 – 23.9 km | 7.88% | 6.20% | 7.07% | 7.82% |
|  | 24 – 30.9 km | 7.66% | 8.09% | 7.27% | 7.82% |
|  | 31 – 100 km or more | 16.22% | 13.21% | 14.95% | 18.87% |
| Household has electricity | Yes | 85.36% | 89.45% | 86.81% | 81.91% |
|  | No | 14.64% | 10.55% | 13.19% | 18.09% |
| Own home | Yes | 48.38% | 46.59% | 46.29% | 54.29% |
|  | No | 51.62% | 53.41% | 53.71% | 45.71% |
| *Infant Developmental Milestones* | | | | | |
| Potty-trained (How often the child defecate into a potty) | Never | 30.99% | 32.08% | 32.91% | 34.65% |
|  | Occasionally | 36.08% | 33.24% | 32.48% | 37.46% |
|  | Mostly | 23.24% | 24.57% | 25.43% | 21.41% |
|  | Always | 9.69% | 10.12% | 9.19% | 6.48% |
| *Family and Health Characteristics through Adolescence* | | | | | |
| Home Location | Urban | 39.87% | 44.27% | 42.11% | 34.74% |
|  | Rural | 60.13% | 55.73% | 57.89% | 65.26% |
| *Educational milestones and Behavior through Adolescence* | | | | | |
| Physical education grades | 7 or below | 26.00% | 25.42% | 25.47% | 36.73% |
|  | 8 | 43.26% | 43.02% | 45.47% | 42.27% |
|  | 9 | 25.30% | 27.65% | 26.95% | 17.20% |
|  | 10 | 5.44% | 3.91% | 2.11% | 3.79% |
| Frequency of sports | Everyday | 21.30% | 28.07% | 22.66% | 19.44% |
|  | Every other day | 24.31% | 24.25% | 27.23% | 23.33% |
|  | Twice a week | 27.31% | 19.89% | 22.87% | 20.28% |
|  | Once a week | 11.34% | 9.26% | 10.19% | 17.78% |
|  | Every second week | 3.01% | 3.81% | 2.29% | 2.22% |
|  | Once a month | 2.78% | 3.54% | 2.70% | 3.06% |
|  | Usually never | 9.95% | 11.17% | 12.06% | 13.89% |
| Smoking | Never tried | 32.10% | 29.00% | 27.05% | 29.04% |
|  | Tried once | 32.56% | 30.62% | 27.05% | 29.32% |
|  | Tried twice or more | 24.25% | 25.75% | 29.71% | 26.85% |
|  | Smoke occasionally | 6.70% | 9.21% | 10.45% | 9.04% |
|  | Smoke twice a week or more | 4.39% | 5.42% | 5.74% | 5.75% |
| Alcohol use | Never drunk any | 46.05% | 38.86% | 40.90% | 41.76% |
|  | Tasted once | 31.40% | 36.41% | 36.20% | 37.36% |
|  | Drank few times, use alcohol monthly or weekly | 22.56% | 24.73% | 22.90% | 20.88% |
| Being drunk | Never | 80.18% | 69.86% | 77.10% | 78.51% |
|  | Once slightly | 8.53% | 12.33% | 10.84% | 8.54% |
|  | Twice or more times slightly | 6.91% | 7.95% | 7.57% | 6.61% |
|  | Once very much | 2.30% | 5.48% | 1.43% | 3.31% |
|  | Several or more times very much | 2.07% | 4.38% | 3.07% | 3.03% |

Note. A total of 54 independent variables were tested for differences between the 4 clusters, separately for both sexes. For males, none of the variables survived correction for multiple comparisons, but those variables on which there were significant differences for females are presented in Supplementary Table 1.
