# Supplementary material for: Early Environment and Neurobehavioral Development Predict Adult Temperament Clusters
Source: PLoS One. 2012 Jul 18;7(7):e38065. doi: 10.1371/journal.pone.0038065 (PMC3399831; doi:10.1371/journal.pone.0038065)
Supplement: Table S3 — Differences in average grades in adolescence between temperament clusters for females and males. (DOC) [file pone.0038065.s003.doc]

Table S3. Differences in average grades in adolescence between temperament clusters for females and males.

|  |  | Temperament Clusters | | | |
| --- | --- | --- | --- | --- | --- |
|  |  | I | II | III | IV |
| *Educational milestones and Behavior through Adolescence* | | | | | |
| Females |  |  |  |  |  |
| Average Grades | Mean | 80.85 | 80.92 | 79.82 | 78.68 |
|  | SD | 7.73 | 7.50 | 7.33 | 7.73 |
|  | N | 512 | 474 | 535 | 404 |
|  | F (3, 1,921) = 8.52, *p* = 1.28 x 10^-5^, R-squared = 0.01 | | | | |
| Males |  |  |  |  |  |
| Average Grades | Mean | 75.17 | 75.12 | 74.17 | 73.53 |
|  | SD | 7.75 | 7.64 | 7.85 | 8.24 |
|  | N | 424 | 359 | 474 | 348 |
|  | F (3, 1,601) = 3.83, *p* = 0.009, R-squared = 0.005 | | | | |
