# Supplementary material for: Early Environment and Neurobehavioral Development Predict Adult Temperament Clusters
Source: PLoS One. 2012 Jul 18;7(7):e38065. doi: 10.1371/journal.pone.0038065 (PMC3399831; doi:10.1371/journal.pone.0038065)
Supplement: Table S4 — Early life measures predicting individual temperament dimensions, as measured by the Temperament and Character Inventory, which survived correction for females. (DOC) [file pone.0038065.s004.doc]

Table S4. Early life measures predicting individual temperament dimensions, as measured by the Temperament and Character Inventory, which survived correction for females.

| *Prenatal Sociodemographic Environment* | |
| --- | --- |
| **Maternal Education*** |  |
| Novelty Seeking | F (4, 1998) = 5.74, p = 1.35 x 10^-4^, R-squared = 0.01, Adjusted R-Squared = 0.01 |
| **Home Location at Birth*** |  |
| Harm Avoidance | F (3, 2031) = 14.29, p = 3.27 x 10^-6^, R-squared = 0.02, Adjusted R-Squared = 0.02 |
| Novelty Seeking | F (3, 2031) = 8.15, p = 2.16 x 10^-5^, R-squared = 0.01, Adjusted R-Squared = 0.01 |
| **Distance to Maternity Clinic*** |  |
| Harm Avoidance | F (6, 1993) = 5.20, p = 2.58 x 10^-5^, R-squared = 0.01, Adjusted R-Squared = 0.01 |
| **Distance to Town Center*** |  |
| Harm Avoidance | F (6, 1979) = 6.03, p = 2.87 x 10^-6^, R-squared = 0.02, Adjusted R-Squared = 0.01 |
| Novelty Seeking | F (6, 1979) = 4.37, p = 2.13 x 10^-4^, R-squared = 0.01, Adjusted R-Squared = 0.01 |
| **Distance to Doctor*** |  |
| Harm Avoidance | F (6, 1972) = 4.86, p = 6.07 x 10^-5^, R-squared = 0.02, Adjusted R-Squared = 0.01 |
| **Own home*** |  |
| Harm Avoidance | F (1, 1955) = 15.09, p = 1.06 x 10^-4^, R-squared = 0.008, Adjusted R-Squared = 0.007 |
| Novelty Seeking | F (1, 1955) = 24.42, p = 8.41 x 10^-7^, R-squared = 0.01, Adjusted R-Squared = 0.01 |
| **Mother worked outside home during pregnancy** |  |
| Novelty Seeking | F (1, 1999) = 14.63, p = 1.35 x 10^-4^, R-squared = 0.007, Adjusted R-Squared = 0.007 |
| *Infant Developmental Milestones* | |
| **Potty-trained*** |  |
| Novelty Seeking | F (3, 1870) = 7.42, p = 6.10 x 10^-5^, R-squared = 0.01, Adjusted R-Squared = 0.01 |
| *Family and Health Characteristics through Adolescence* | |
| **Place of residence at adolescence*** |  |
| Harm Avoidance | F (1, 2033) = 24.68, p = 7.34 x 10^-7^, R-squared = 0.01, Adjusted R-Squared = 0.01 |
| Novelty Seeking | F (1, 2033) = 23.21, p = 1.56 x 10^-6^, R-squared = 0.01, Adjusted R-Squared = 0.01 |
| *Educational milestones and Behavior through Adolescence* | |
| **Admitted to high-school** |  |
| Harm Avoidance | F (1, 1989) = 16.37, p = 5.40 x 10^-5^, R-squared = 0.008, Adjusted R-Squared = 0.008 |
| **Type of high-school admitted to** |  |
| Harm Avoidance | F (3, 2031) = 6.78, p = 1.51 x 10^-4^, R-squared = 0.01, Adjusted R-Squared = 0.008 |
| **Average Grades*** |  |
| Harm Avoidance | F (1, 1923) = 24.93, p = 6.48 x 10^-7^, R-squared = 0.01, Adjusted R-Squared = 0.01 |
| Persistence | F (1, 1923) = 34.65, p = 4.64 x 10^-9^, R-squared = 0.02, Adjusted R-Squared = 0.02 |
| **Physical Education Grades*** |  |
| Harm Avoidance | F (3, 1932) = 17.33, p = 4.18 x 10^-11^, R-squared = 0.03, Adjusted R-Squared = 0.02 |
| **Sport Frequency*** |  |
| Harm Avoidance | F (6, 1947) = 8.60, p = 2.99 x 10^-9^, R-squared = 0.03, Adjusted R-Squared = 0.02 |
| **Smoking*** |  |
| Novelty Seeking | F (4, 1968) = 10.74, p = 1.31 x 10^-8^, R-squared = 0.02, Adjusted R-Squared = 0.02 |
| **Drinking*** |  |
| Novelty Seeking | F (2, 1970) = 21.20, p = 7.80 x 10^-10^, R-squared = 0.02, Adjusted R-Squared = 0.02 |
| **Drunkenness*** |  |
| Novelty Seeking | F (4, 1965) = 14.61, p = 9.11 x 10^-12^, R-squared = 0.03, Adjusted R-Squared = 0.03 |

Note: A total of 54 independent variables were tested as predictors of the four separate TCI. The differences that remained significant after Bonferroni correction (p < 0.00023) are presented. *Indicates those variables that also significantly differed between temperament clusters after correction.
