# Supplementary material for: Early Environment and Neurobehavioral Development Predict Adult Temperament Clusters
Source: PLoS One. 2012 Jul 18;7(7):e38065. doi: 10.1371/journal.pone.0038065 (PMC3399831; doi:10.1371/journal.pone.0038065)
Supplement: Table S5 — Early life measures predicting individual temperament dimensions, as measured by the Temperament and Character Inventory, which survived correction for males. (DOC) [file pone.0038065.s005.doc]

Table S5. Early life measures predicting individual temperament dimensions, as measured by the Temperament and Character Inventory, which survived correction for males.

| *Educational milestones and Behavior through Adolescence* | |
| --- | --- |
| **School level classification** |  |
| Harm Avoidance | F (1, 1724) = 13.82, p = 2.07 x 10^-4^, R-squared = 0.008, Adjusted R-Squared = 0.007 |
| **Type of high-school admitted to** |  |
| Harm Avoidance | F (3, 1722) = 7.21, p = 8.23 x 10^-5^, R-squared = 0.01, Adjusted R-Squared = 0.01 |
| **Average Grades*** |  |
| Harm Avoidance | F (1, 1603) = 17.36, p = 3.26 x 10^-5^, R-squared = 0.01, Adjusted R-Squared = 0.01 |
| **Physical Education Grades*** |  |
| Harm Avoidance | F (3, 1595) = 7.65, p = 4.43 x 10^-5^, R-squared = 0.01, Adjusted R-Squared = 0.01 |
| **Sport Frequency*** |  |
| Reward Dependence | F (6, 1633) = 4.60, p = 1.21 x 10^-4^, R-squared = 0.02, Adjusted R-Squared = 0.01 |
| **Drunkenness*** |  |
| Novelty Seeking | F (4, 1646) = 7.99, p = 2.25 x 10^-6^, R-squared = 0.02, Adjusted R-Squared = 0.02 |

Note: A total of 54 independent variables were tested as predictors of the four separate TCI scale. The differences that remained significant after Bonferroni correction (p < 0.00023) are presented. *Indicates those variables that also significantly differed between temperament clusters after correction.
