# Supplementary material for: Early Environment and Neurobehavioral Development Predict Adult Temperament Clusters
Source: PLoS One. 2012 Jul 18;7(7):e38065. doi: 10.1371/journal.pone.0038065 (PMC3399831; doi:10.1371/journal.pone.0038065)
Supplement: Table S6 — Early life measures predicting group membership of each female temperament cluster separately. (DOC) [file pone.0038065.s006.doc]

Table S6. Early life measures predicting group membership of each female temperament cluster separately.

|  |  | **Regression Coefficient** | ***p*** | **OR** | **Lower CI** | **Upper CI** |
| --- | --- | --- | --- | --- | --- | --- |
| **Cluster I** | | | | | | |
| Maternal education |  |  |  |  |  |  |
|  | No or 1-4 years primary school | -- | -- | -- | -- | -- |
|  | 5-8 years or unfinished primary school | -0.30 | 0.14 | 0.74 | 0.49 | 1.11 |
|  | Some or over 2 years vocational school | -0.58 | 0.02 | 0.56 | 0.34 | 0.90 |
|  | 5 or more years secondary school | -0.51 | 0.07 | 0.60 | 0.34 | 1.05 |
|  | Matriculation or more | -0.96 | 0.02 | 0.38 | 0.17 | 0.84 |
| Mother lived in same region entire life | Yes or No | 0.32 | 0.02 | 1.37 | 1.06 | 1.78 |
| Home location at birth |  |  |  |  |  |  |
|  | City | -- | -- | -- | -- | -- |
|  | Small town | 0.47 | 0.17 | 1.60 | 0.81 | 3.17 |
|  | Rural center | -0.29 | 0.11 | 0.75 | 0.53 | 1.07 |
|  | Remote village | -0.03 | 0.88 | 0.97 | 0.61 | 1.52 |
| Distance to doctor |  |  |  |  |  |  |
|  | Less than 300 m | -- | -- | -- | -- | -- |
|  | 300 m – 2.9 km | -0.58 | 0.01 | 0.56 | 0.36 | 0.88 |
|  | 3 – 9.9 km | -0.65 | 0.01 | 0.52 | 0.31 | 0.88 |
|  | 10 – 16.9 km | -0.53 | 0.08 | 0.59 | 0.32 | 1.07 |
|  | 17 – 23.9 km | -0.10 | 0.76 | 0.90 | 0.46 | 1.77 |
|  | 24 – 30.9 km | -0.58 | 0.10 | 0.56 | 0.28 | 1.12 |
|  | 31 – 100 km or more | -0.80 | 0.01 | 0.45 | 0.24 | 0.85 |
| Mother’s frame of mind during the pregnancy | As usual or Depressed/Very depressed | 0.28 | 0.10 | 1.32 | 0.94 | 1.86 |
| Admitted to secondary school | No or Yes | -0.27 | 0.10 | 0.76 | 0.55 | 1.06 |
| Average grades in adolescence |  | 0.04 | 0.0005 | 1.04 | 1.02 | 1.06 |
| Smoking in adolescence |  |  |  |  |  |  |
|  | Never tried | -- | -- | -- | -- | -- |
|  | Tried once | -0.27 | 0.08 | 0.76 | 0.56 | 1.03 |
|  | Tried twice or more | -0.47 | 0.003 | 0.62 | 0.45 | 0.85 |
|  | Smoke occasionally | -0.86 | 0.0008 | 0.43 | 0.26 | 0.70 |
|  | Smoke twice a week or more | -0.61 | 0.03 | 0.54 | 0.31 | 0.94 |
|  | Generalized R-Squared = 0.04 | | | | | |
| **Cluster II** | | | | | | |
| Mother’s age |  | -0.03 | 0.005 | 0.97 | 0.95 | 0.99 |
| Household has running water | Yes or No | -0.26 | 0.04 | 0.77 | 0.60 | 0.99 |
| Number of words spoken by age one |  |  |  |  |  |  |
|  | Zero | -- | -- | -- | -- | -- |
|  | One | -0.30 | 0.15 | 0.74 | 0.49 | 1.11 |
|  | Two | 0.15 | 0.37 | 1.16 | 0.84 | 1.61 |
|  | Three or more | 1.09 | 0.002 | 2.99 | 1.50 | 5.96 |
| Physical education grades in adolescence |  |  |  |  |  |  |
|  | 7 or lower | -- | -- | -- | -- | -- |
|  | 8 | 0.26 | 0.12 | 1.30 | 0.94 | 1.81 |
|  | 9 | 0.53 | 0.003 | 1.70 | 1.20 | 2.43 |
|  | 10 | 0.97 | 0.003 | 2.63 | 1.38 | 5.02 |
| Smoking in adolescence |  |  |  |  |  |  |
|  | Never | -- | -- | -- | -- | -- |
|  | Tried once | 0.17 | 0.31 | 1.19 | 0.85 | 1.66 |
|  | Tried twice or more | 0.22 | 0.19 | 1.25 | 0.90 | 1.74 |
|  | Smoke occasionally | 0.83 | 0.0002 | 2.30 | 1.49 | 3.55 |
|  | Smoke twice a week or more | 0.88 | 0.0003 | 2.42 | 1.50 | 3.90 |
|  | Generalized R-Squared = 0.04 | | | | | |
| **Cluster III** | | | | | | |
| Maternal education |  |  |  |  |  |  |
|  | No or 1-4 years primary school | -- | -- | -- | -- | -- |
|  | 5-8 years or unfinished primary school | 0.46 | 0.05 | 1.59 | 1.00 | 2.51 |
|  | Some or over 2 years vocational school | 0.82 | 0.002 | 2.27 | 1.37 | 3.78 |
|  | 5 or more years secondary school | 0.80 | 0.01 | 2.23 | 1.26 | 3.95 |
|  | Matriculation or more | 0.89 | 0.02 | 2.44 | 1.14 | 5.25 |
| Height in adolescence |  | 0.02 | 0.06 | 1.02 | 1.00 | 1.04 |
| Average grades in adolescence |  | -0.02 | 0.02 | 0.98 | 0.96 | 1.00 |
| Smoking in adolescence |  |  |  |  |  |  |
|  | Never | -- | -- | -- | -- | -- |
|  | Tried once | 0.56 | 0.0005 | 1.75 | 1.27 | 2.40 |
|  | Tried twice or more | 0.56 | 0.0006 | 1.74 | 1.27 | 2.40 |
|  | Smoke occasionally | 0.51 | 0.02 | 1.66 | 1.07 | 2.58 |
|  | Smoke twice a week or more | 0.30 | 0.25 | 1.35 | 0.81 | 2.25 |
|  | Generalized R-Squared = 0.03 | | | | | |
| **Cluster IV** | | | | | | |
| Family owns home at birth | Yes or No | -0.36 | 0.01 | 0.70 | 0.52 | 0.93 |
| Father’s occupation in adolescence | Unskilled or Skilled | 0.48 | 0.10 | 1.61 | 0.91 | 2.85 |
| Home location in adolescence | Urban or Rural | 0.40 | 0.15 | 1.50 | 1.11 | 2.02 |
| Height in adolescence |  | -0.02 | 0.05 | 0.98 | 0.96 | 1.00 |
| School admission |  |  |  |  |  |  |
|  | Reached secondary school | -- | -- | -- | -- | -- |
|  | Reached vocational school | 0.13 | 0.51 | 1.13 | 0.78 | 1.64 |
|  | Reached both secondary and vocational school | 0.18 | 0.35 | 1.19 | 0.83 | 1.72 |
|  | Applied but wasn't admitted or didn't apply | -1.73 | 0.10 | 0.18 | 0.02 | 1.38 |
| Average grades in adolescence |  | -0.02 | 0.05 | 0.98 | 0.96 | 1.00 |
| Frequency of sports outside of school |  |  |  |  |  |  |
|  | Everyday | -- | -- | -- | -- | -- |
|  | Every other day | 0.33 | 0.26 | 1.39 | 0.79 | 2.44 |
|  | Twice a week | 0.30 | 0.25 | 1.36 | 0.81 | 2.28 |
|  | Once a week | 0.81 | 0.002 | 2.25 | 1.35 | 3.75 |
|  | Every second week | 0.56 | 0.15 | 1.75 | 0.81 | 3.77 |
|  | Once a month | 0.19 | 0.64 | 1.21 | 0.54 | 2.69 |
|  | Usually never | 0.94 | 0.0003 | 2.57 | 1.53 | 4.29 |
| Smoking in adolescence |  |  |  |  |  |  |
|  | Never | -- | -- | -- | -- | -- |
|  | Tried once | -0.54 | 0.003 | 0.58 | 0.41 | 0.83 |
|  | Tried twice or more | -0.42 | 0.02 | 0.66 | 0.47 | 0.93 |
|  | Smoke occasionally | -0.57 | 0.03 | 0.57 | 0.34 | 0.96 |
|  | Smoke twice a week or more | -0.90 | 0.01 | 0.41 | 0.22 | 0.77 |
|  | Generalized R-Squared = 0.09 | | | | | |

Note: For each predictor variable with more than two levels, the first level was used as the reference category in the regression analyses; OR: odds ratio (the exponentiated regression coefficient); CI: 95% confidence intervals of the odds ratio. The sample sizes for each of the analyses, after eliminating any individual with missing values on any of the predictor variables, were: I = 392, II = 355, III = 385, and IV = 295 (total N = 1427).
