# Supplementary material for: Early Environment and Neurobehavioral Development Predict Adult Temperament Clusters
Source: PLoS One. 2012 Jul 18;7(7):e38065. doi: 10.1371/journal.pone.0038065 (PMC3399831; doi:10.1371/journal.pone.0038065)
Supplement: Table S7 — Early life measures predicting group membership of each male temperament cluster separately. (DOC) [file pone.0038065.s007.doc]

Table S7. Early life measures predicting group membership of each male temperament cluster separately.

|  |  | **Regression Coefficient** | ***p*** | **OR** | **Lower CI** | **Upper CI** |
| --- | --- | --- | --- | --- | --- | --- |
| **Cluster I** | | | | | | |
| Maternal education |  |  |  |  |  |  |
|  | No or 1-4 years primary school | -- | -- | -- | -- | -- |
|  | 5-8 years or unfinished primary school | -0.40 | 0.04 | 0.67 | 0.45 | 0.99 |
|  | Some or over 2 years vocational school | -0.27 | 0.22 | 0.76 | 0.49 | 1.18 |
|  | 5 or more years secondary school | -0.75 | 0.006 | 0.47 | 0.28 | 0.80 |
|  | Matriculation or more | -0.15 | 0.64 | 0.86 | 0.45 | 1.63 |
| Times applied to secondary school |  |  |  |  |  |  |
|  | Zero | -- | -- | -- | -- | -- |
|  | Once | 0.24 | 0.53 | 1.27 | 0.60 | 2.73 |
|  | Twice or more | 0.50 | 0.21 | 1.64 | 0.76 | 3.57 |
| Average grades in adolescence |  | 0.02 | 0.02 | 1.02 | 1.00 | 1.03 |
|  | Generalized R-Squared = 0.01 | | | | | |
| **Cluster II** | | | | | | |
| Maternal education |  |  |  |  |  |  |
|  | No or 1-4 years primary school | -- | -- | -- | -- | -- |
|  | 5-8 years or unfinished primary school | 0.62 | 0.04 | 1.85 | 1.04 | 3.30 |
|  | Some or over 2 years vocational school | 0.79 | 0.01 | 2.19 | 1.19 | 4.05 |
|  | 5 or more years secondary school | 0.91 | 0.006 | 2.49 | 1.29 | 4.80 |
|  | Matriculation or more | 0.58 | 0.15 | 1.79 | 0.80 | 4.01 |
| Mother’s age |  | -0.02 | 0.15 | 0.98 | 0.96 | 1.01 |
| Mother exposed to outside information during pregnancy |  |  |  |  |  |  |
|  | Regularly | -- | -- | -- | -- | -- |
|  | Fairly often | -0.41 | 0.06 | 0.67 | 0.43 | 1.02 |
|  | Occasionally | -0.68 | 0.002 | 0.51 | 0.33 | 0.78 |
|  | Seldom or never | -0.78 | 0.008 | 0.46 | 0.26 | 0.81 |
| Being drunk in adolescence |  |  |  |  |  |  |
|  | Never | -- | -- | -- | -- | -- |
|  | Once slightly | 0.30 | 0.14 | 1.35 | 0.90 | 2.03 |
|  | Twice or more times slightly | 0.23 | 0.35 | 1.26 | 0.78 | 2.04 |
|  | Once very much | 0.97 | 0.003 | 2.64 | 1.41 | 4.95 |
|  | Several or more times very much | 0.59 | 0.07 | 1.80 | 0.95 | 3.40 |
|  | Generalized R-Squared = 0.03 | | | | | |
| **Cluster III** | | | | | | |
| Primary parent occupation at birth | Unskilled or Skilled | 0.25 | 0.12 | 1.28 | 0.94 | 1.73 |
| Mother’s age |  | 0.02 | 0.01 | 1.02 | 1.00 | 1.04 |
| Mother worked outside of home during pregnancy | No or Yes | 0.20 | 0.12 | 1.22 | 0.95 | 1.56 |
| Desirability of the pregnancy |  |  |  |  |  |  |
|  | Occurred at a propitious time | -- | -- | -- | -- | -- |
|  | Would have been more desirable later | -0.19 | 0.19 | 0.83 | 0.62 | 1.10 |
|  | Should not have occurred at all | -0.51 | 0.02 | 0.60 | 0.40 | 0.91 |
| Times applied to secondary school |  |  |  |  |  |  |
|  | Zero | -- | -- | -- | -- | -- |
|  | Once | 0.39 | 0.28 | 1.48 | 0.72 | 3.03 |
|  | Twice or more | 0.00 | 0.99 | 1.00 | 0.48 | 2.09 |
| Average grades in adolescence |  | -0.02 | 0.02 | 0.98 | 0.97 | 1.00 |
| Physical education grades in adolescence |  |  |  |  |  |  |
|  | 7 or lower | -- | -- | -- | -- | -- |
|  | 8 | 0.23 | 0.11 | 1.26 | 0.95 | 1.68 |
|  | 9 | 0.37 | 0.03 | 1.45 | 1.04 | 2.02 |
|  | 10 | -0.35 | 0.36 | 0.71 | 0.34 | 1.49 |
|  | Generalized R-Squared = 0.03 | | | | | |
| **Cluster IV** | | | | | | |
| Desirability of the pregnancy |  |  |  |  |  |  |
|  | Occurred at a propitious time | -- | -- | -- | -- | -- |
|  | Would have been more desirable later | 0.43 | 0.005 | 1.54 | 1.14 | 2.08 |
|  | Should not have occurred at all | 0.41 | 0.04 | 1.51 | 1.01 | 2.25 |
| Home location in adolescence | Urban or Rural | 0.28 | 0.05 | 1.32 | 1.00 | 1.75 |
| School level classification | Below or Above median | -0.84 | 0.007 | 0.43 | 0.24 | 0.79 |
| Times applied to secondary school |  |  |  |  |  |  |
|  | Zero | -- | -- | -- | -- | -- |
|  | Once | -0.81 | 0.01 | 0.45 | 0.23 | 0.85 |
|  | Twice or more | -0.72 | 0.03 | 0.49 | 0.25 | 0.95 |
| Physical education grades in adolescence |  |  |  |  |  |  |
|  | 7 or lower | -- | -- | -- | -- | -- |
|  | 8 | -0.29 | 0.05 | 0.75 | 0.55 | 1.01 |
|  | 9 | -0.67 | 0.0004 | 0.51 | 0.35 | 0.74 |
|  | 10 | -0.51 | 0.19 | 0.60 | 0.28 | 1.28 |
|  | Generalized R-Squared = 0.05 | | | | | |

Note: For each predictor variable with more than two levels, the first level was used as the reference category in the regression analyses; OR: odds ratio (the exponentiated regression coefficient); CI: 95% confidence intervals of the odds ratio. The sample sizes for each of the analyses, after eliminating any individual with missing values on any of the predictor variables, were: I = 385, II = 326, III = 430, and IV = 300 (total N = 1441).
