# Supplementary material for: Early Environment and Neurobehavioral Development Predict Adult Temperament Clusters
Source: PLoS One. 2012 Jul 18;7(7):e38065. doi: 10.1371/journal.pone.0038065 (PMC3399831; doi:10.1371/journal.pone.0038065)
Supplement: Table S8 — Early life measures predicting temperament dimension scores for females. (DOC) [file pone.0038065.s008.doc]

Table S8. Early life measures predicting temperament dimension scores for females.

|  |  | **Regression Coefficient** | ***p*** | **Lower CI** | **Upper CI** |
| --- | --- | --- | --- | --- | --- |
| **Harm Avoidance** | | | | | |
| Distance to doctor |  |  |  |  |  |
|  | Less than 300 m | -- | -- | -- | -- |
|  | 300 m – 2.9 km | 1.10 | 0.08 | -0.15 | 2.35 |
|  | 3 – 9.9 km | 1.35 | 0.05 | -0.02 | 2.72 |
|  | 10 – 16.9 km | 1.96 | 0.01 | 0.43 | 3.49 |
|  | 17 – 23.9 km | 0.55 | 0.54 | -1.23 | 2.34 |
|  | 24 – 30.9 km | 2.22 | 0.01 | 0.48 | 3.96 |
|  | 31 – 100 km or more | 2.72 | 0.00 | 1.28 | 4.16 |
| Family owns home at birth | Yes or No | -0.58 | 0.13 | -1.32 | 0.17 |
| Mother worked outside of home during pregnancy | No or Yes | 0.63 | 0.10 | -0.13 | 1.38 |
| Number of words spoken by age one |  |  |  |  |  |
|  | Zero | -- | -- | -- | -- |
|  | One | 0.83 | 0.11 | -0.19 | 1.84 |
|  | Two | 0.02 | 0.96 | -0.84 | 0.89 |
|  | Three or more | -1.69 | 0.11 | -3.76 | 0.39 |
| Child wets self during the day at age one |  |  |  |  |  |
|  | Every day | -- | -- | -- | -- |
|  | Hardly ever | -0.78 | 0.04 | -1.51 | -0.05 |
|  | Never | -0.35 | 0.51 | -1.41 | 0.71 |
| Father’s occupation in adolescence | Unskilled or Skilled | 0.80 | 0.15 | -0.29 | 1.89 |
| Physical education grades in adolescence |  |  |  |  |  |
|  | 7 or lower | -- | -- | -- | -- |
|  | 8 | -0.92 | 0.03 | -1.75 | -0.09 |
|  | 9 | -1.75 | 0.00 | -2.69 | -0.81 |
|  | 10 | -2.31 | 0.02 | -4.20 | -0.41 |
| Frequency of sports outside of school |  |  |  |  |  |
|  | Everyday | -- | -- | -- | -- |
|  | Every other day | 0.59 | 0.36 | -0.66 | 1.83 |
|  | Twice a week | 0.85 | 0.14 | -0.28 | 1.98 |
|  | Once a week | 1.86 | 0.00 | 0.70 | 3.02 |
|  | Every second week | 1.24 | 0.18 | -0.58 | 3.06 |
|  | Once a month | 1.78 | 0.06 | -0.08 | 3.64 |
|  | Usually never | 2.80 | 0.00 | 1.60 | 3.99 |
| Drinking in adolescence |  |  |  |  |  |
|  | Never | -- | -- | -- | -- |
|  | Tasted once | -0.38 | 0.33 | -1.14 | 0.39 |
|  | Drunken few times or more | -0.93 | 0.03 | -1.75 | -0.10 |
|  | R-Squared = 0.07, Adjusted R-Squared = 0.05 | | | | |
| **Novelty Seeking** | | | | | |
| Mother’s age |  | -0.07 | 0.01 | -0.12 | -0.02 |
| Home location at birth |  |  |  |  |  |
|  | City | -- | -- | -- | -- |
|  | Small town | -1.26 | 0.22 | -3.27 | 0.76 |
|  | Rural center | 1.23 | 0.03 | 0.15 | 2.31 |
|  | Remote village | 0.51 | 0.33 | -0.52 | 1.54 |
| Mother exposed to outside information during pregnancy |  |  |  |  |  |
|  | Regularly | -- | -- | -- | -- |
|  | Fairly often | -1.34 | 0.03 | -2.55 | -0.13 |
|  | Occasionally | -1.83 | 0.00 | -3.03 | -0.63 |
|  | Seldom or never | -2.16 | 0.00 | -3.65 | -0.67 |
| Number of words spoken by age one |  |  |  |  |  |
|  | Zero | -- | -- | -- | -- |
|  | One | -0.43 | 0.44 | -1.51 | 0.66 |
|  | Two | -0.36 | 0.44 | -1.28 | 0.56 |
|  | Three or more | 2.77 | 0.01 | 0.76 | 4.77 |
| Child wets self during the night at age one | Every day or Hardly ever/Never | 0.71 | 0.08 | -0.08 | 1.51 |
| Father’s occupation in adolescence | Unskilled or Skilled | -1.41 | 0.02 | -2.58 | -0.25 |
| Home location in adolescence | Urban or Rural | -0.75 | 0.10 | -1.64 | 0.14 |
| Smoking in adolescence |  |  |  |  |  |
|  | Never | -- | -- | -- | -- |
|  | Tried once | 0.46 | 0.34 | -0.49 | 1.40 |
|  | Tried twice or more | 0.61 | 0.24 | -0.41 | 1.62 |
|  | Smoke occasionally | 2.56 | 0.00 | 1.06 | 4.05 |
|  | Smoke twice a week or more | 2.79 | 0.00 | 1.12 | 4.46 |
| Drinking in adolescence |  |  |  |  |  |
|  | Never | -- | -- | -- | -- |
|  | Tasted once | 1.10 | 0.01 | 0.23 | 1.97 |
|  | Drunken few times or more | 0.63 | 0.27 | -0.50 | 1.76 |
|  | R-Squared = 0.06, Adjusted R-Squared = 0.05 | | | | |
| **Reward Dependence** | | | | | |
| Mother lived in same region entire life | Yes or No | 0.51 |  | 0.13 | 0.90 |
| Household has electricity at birth | Yes or No | -0.76 |  | -1.29 | -0.23 |
| School admission |  |  |  |  |  |
|  | Reached secondary school | -- | -- | -- | -- |
|  | Reached vocational school | -0.23 |  | -0.65 | 0.18 |
|  | Reached both secondary and vocational school | -0.49 |  | -1.01 | 0.02 |
|  | Applied but wasn't admitted or didn't apply | 1.80 |  | 0.41 | 3.19 |
|  | R-Squared = 0.02, Adjusted R-Squared = 0.01 | | | | |
| **Persistence** | | | | | |
| Height at birth |  | -0.03 | 0.15 | -0.08 | 0.01 |
| Average grades |  | 0.03 | 0.00 | 0.02 | 0.04 |
|  | R-Squared = 0.02, Adjusted R-Squared = 0.02 | | | | |

Note: For each predictor variable with more than two levels, the first level was used as the reference category in the regression analyses; CI: 95% confidence intervals of the regression coefficient. The sample sizes for each of the analyses, after eliminating any individual with missing values on any of the predictor variables, were: HA = 1189, NS = 1178, RD = 1269, and P = 1345.
