# Supplementary material for: Early Environment and Neurobehavioral Development Predict Adult Temperament Clusters
Source: PLoS One. 2012 Jul 18;7(7):e38065. doi: 10.1371/journal.pone.0038065 (PMC3399831; doi:10.1371/journal.pone.0038065)
Supplement: Table S9 — Early life measures predicting temperament dimension scores for males. (DOC) [file pone.0038065.s009.doc]

Table S9. Early life measures predicting temperament dimension scores for males.

|  |  | **Regression Coefficient** | ***p*** | **Lower CI** | **Upper CI** |
| --- | --- | --- | --- | --- | --- |
| **Harm Avoidance** | | | | | |
| Mother’s age |  | 0.07 | 0.03 | 0.01 | 0.12 |
| Distance to neighbor | Less than 300 m or 300 m or more | 1.03 | 0.12 | -0.27 | 2.33 |
| Family owns car at birth | Yes or No | 0.94 | 0.02 | 0.14 | 1.75 |
| Mother exposed to outside information during pregnancy |  |  |  |  |  |
|  | Regularly | -- | -- | -- | -- |
|  | Fairly often | 0.37 | 0.62 | -1.12 | 1.87 |
|  | Occasionally | 0.96 | 0.20 | -0.51 | 2.43 |
|  | Seldom or never | 1.95 | 0.03 | 0.16 | 3.74 |
| Child wets self during the day at age one |  |  |  |  |  |
|  | Every day | -- | -- | -- | -- |
|  | Hardly ever | -0.59 | 0.19 | -1.47 | 0.30 |
|  | Never | -1.95 | 0.01 | -3.34 | -0.55 |
| School level classification | Below vs Above median | -1.89 | 0.13 | -4.31 | 0.54 |
| Frequency of sports outside of school |  |  |  |  |  |
|  | Everyday | -- | -- | -- | -- |
|  | Every other day | 1.08 | 0.06 | -0.03 | 2.20 |
|  | Twice a week | 0.34 | 0.56 | -0.81 | 1.49 |
|  | Once a week | 2.29 | 0.00 | 0.84 | 3.74 |
|  | Every second week | -1.47 | 0.25 | -3.98 | 1.04 |
|  | Once a month | 2.58 | 0.05 | 0.01 | 5.15 |
|  | Usually never | 1.28 | 0.08 | -0.13 | 2.69 |
| Intoxicant use in adolescence | No or Yes | -5.61 | 0.02 | -10.40 | -0.82 |
|  | R-Squared = 0.05, Adjusted R-Squared = 0.03 | | | | |
| **Novelty Seeking** | | | | | |
| Household has electricity at birth | Yes or No | -0.87 | 0.13 | -2.00 | 0.27 |
| Father’s occupation in adolescence | Unskilled or Skilled | -1.10 | 0.06 | -2.27 | 0.06 |
| Being drunk in adolescence |  |  |  |  |  |
|  | Never | -- | -- | -- | -- |
|  | Once slightly | 2.34 | 0.00 | 1.04 | 3.64 |
|  | Twice or more times slightly | 1.11 | 0.13 | -0.31 | 2.53 |
|  | Once very much | 0.40 | 0.73 | -1.86 | 2.67 |
|  | Several or more times very much | 2.07 | 0.06 | -0.12 | 4.25 |
| Intoxicant use in adolescence | No or Yes | 6.56 | 0.02 | 1.20 | 11.91 |
|  | R-Squared = 0.02, Adjusted R-Squared = 0.02 | | | | |
| **Reward Dependence** | | | | | |
| Mother lived in same region entire life | Yes or No |  |  |  |  |
| Distance to town center |  |  |  |  |  |
|  | Less than 300 m | -- | -- | -- | -- |
|  | 300 m – 2.9 km | 0.21 | 0.71 | -0.90 | 1.31 |
|  | 3 – 9.9 km | 0.22 | 0.66 | -0.77 | 1.21 |
|  | 10 – 16.9 km | -1.17 | 0.06 | -2.39 | 0.05 |
|  | 17 – 23.9 km | -0.61 | 0.48 | -2.27 | 1.06 |
|  | 24 – 30.9 km | -3.30 | 0.00 | -5.03 | -1.57 |
|  | 31 – 100 km or more | -0.36 | 0.65 | -1.92 | 1.21 |
| Distance to doctor |  |  |  |  |  |
|  | Less than 300 m | -- | -- | -- | -- |
|  | 300 m – 2.9 km | 0.75 | 0.11 | -0.17 | 1.67 |
|  | 3 – 9.9 km | 0.67 | 0.25 | -0.47 | 1.81 |
|  | 10 – 16.9 km | 1.51 | 0.03 | 0.15 | 2.87 |
|  | 17 – 23.9 km | 0.62 | 0.46 | -1.01 | 2.25 |
|  | 24 – 30.9 km | 3.61 | 0.00 | 1.84 | 5.38 |
|  | 31 – 100 km or more | 0.42 | 0.62 | -1.25 | 2.09 |
| Number of words spoken by age one |  |  |  |  |  |
|  | Zero | -- | -- | -- | -- |
|  | One | 0.09 | 0.80 | -0.60 | 0.78 |
|  | Two | 0.66 | 0.02 | 0.10 | 1.21 |
|  | Three or more | -2.30 | 0.02 | -4.24 | -0.36 |
| Child wets self during the day at age one |  |  |  |  |  |
|  | Every day | -- | -- | -- | -- |
|  | Hardly ever | 0.52 | 0.06 | -0.01 | 1.05 |
|  | Never | 0.57 | 0.19 | -0.29 | 1.42 |
| School admission |  |  |  |  |  |
|  | Reached secondary school | -- | -- | -- | -- |
|  | Reached vocational school | -0.62 | 0.02 | -1.16 | -0.09 |
|  | Reached both secondary and vocational school | -1.30 | 0.02 | -2.37 | -0.23 |
|  | Applied but wasn't admitted or didn't apply | -0.46 | 0.67 | -2.60 | 1.67 |
| Times applied to secondary school | Never/Once or Twice or more | 0.75 | 0.01 | 0.16 | 1.34 |
| Repeated grade in school | Never or Once or more | 1.15 | 0.16 | -0.44 | 2.73 |
|  | R-Squared = 0.04, Adjusted R-Squared = 0.02 | | | | |
| **Persistence** | | | | | |
| Maternal education |  |  |  |  |  |
|  | No or 1-4 years primary school | -- | -- | -- | -- |
|  | 5-8 years or unfinished primary school | 0.54 | 0.01 | 0.16 | 0.91 |
|  | Some or over 2 years vocational school | 0.64 | 0.00 | 0.22 | 1.07 |
|  | 5 or more years secondary school | 0.54 | 0.02 | 0.07 | 1.01 |
|  | Matriculation or more | 0.59 | 0.06 | -0.02 | 1.21 |
|  | R-Squared = 0.007, Adjusted R-Squared = 0.005 | | | | |

Note: For each predictor variable with more than two levels, the first level was used as the reference category in the regression analyses; CI: 95% confidence intervals of the regression coefficient. The sample sizes for each of the analyses, after eliminating any individual with missing values on any of the predictor variables, were: HA = 890, NS = 923, RD = 947, and P = 1025.
